# Single vs Double Lung Transplantation in Older Adults: A Propensity-Matched Analysis

**DOI:** 10.1016/j.chest.2024.08.044

**Published:** 2024-09-05

**Authors:** Noah Weingarten, Atul C. Mehta, Marie Budev, Usman Ahmad, James Yun, Kenneth McCurry, Haytham Elgharably

**Affiliations:** a Department of Surgery, University of Pennsylvania, Philadelphia, PA; b Heart, Vascular and Thoracic Institute, Department of Cardiovascular Surgery, Cleveland Clinic, Cleveland, OH; c Respiratory Institute, Department of Pulmonary Medicine, Cleveland Clinic, Cleveland, OH

**Keywords:** bilateral lung transplantation, older adult, outcomes, single lung transplantation

## Abstract

**Background:**

Single lung transplantation (SLT) has been shown to be associated with worse long-term outcomes than bilateral lung transplantation (BLT), but often is performed in older adults at risk of not tolerating BLT.

**Research Question:**

How do the outcomes of SLT and BLT compare among older adult recipients?

**Study Design and Methods:**

The Scientific Registry of Transplant Recipients database (2005-2022) was queried for lung transplant recipients aged 65 years older. Patients were stratified by whether they underwent BLT or SLT and were propensity matched. Baseline characteristics and morbidity were compared with frequentist statistics. Survival was analyzed via Kaplan-Meier estimation. Risk factors for mortality were identified with Cox regression.

**Results:**

Of 9,904 included patients, 4,829 patients (48.8%) underwent SLT. Patients who underwent SLT had lower lung allocation scores (39.6 vs 40.6; *P* < .001), more interstitial lung disease (74.4% vs 64.6%; *P* < .001), and lower rates of bridging (0.7% vs 2.4%; *P* < .001). Groups did not differ significantly by sex, BMI, or donor characteristics. Propensity matching resulted in 2,539 patients in each group. On matched analysis, patients undergoing SLT had shorter lengths of stay (14 days vs 18 day), lower reintubation rates (14.7% vs 19.8%), and less postoperative dialysis use (4.2% vs 6.4%; *P* < .001 for all). Patients who underwent SLT had comparable survival at 30 days (97.6% vs 97.3%; *P* = .414) and 1 year (85.5% vs 86.3%; *P* = .496), but lower survival at 5 years (45.4% vs 53.4%; *P* < .001) on matched analysis. SLT was a risk factor for 5-year mortality (adjusted hazard ratio, 1.19; *P* < .001).

**Interpretation:**

Our findings show that in older adults, SLT is associated with less morbidity and comparable early survival relative to BLT, but lower 5-year survival. This suggests that SLT is reasonable to perform in older adults at high risk of not tolerating BLT.

FOR EDITORIAL COMMENT, SEE PAGE 312

**Take-home Points**

**Study Question:** Among patients aged 65 years or older, how do outcomes after single and bilateral lung transplantation compare?

**Results:** Compared with recipients of bilateral lung transplantation, recipients of single lung transplantation had shorter lengths of stay and less postoperative dialysis use, as well as comparable 30-day and 1-year survival and slightly lower 5-year survival.

**Interpretation:** Given its relatively low postoperative morbidity, comparable early mortality, and slightly worse long-term mortality, single lung transplant remains reasonable to perform in select older adult patients.

Bilateral lung transplantation (BLT) is associated with greater long-term survival than single lung transplantation (SLT).^1^, ^2^, ^3^ In retrospective analyses, BLT recipients have been found to have improved pulmonary function and exercise tolerance,^4^^,^^5^ greater quality of life,^6^ and lower risk of bronchiolitis obliterans syndrome than SLT recipients.^7^ As a result, the relative ratio of BLT to SLT performed in the United States has nearly doubled over the last decade.^8^

Yet on a population level, SLT offers the potential to dramatically increase the number of patients receiving transplants, thereby reducing waitlist times and waitlist-associated morbidity and mortality.^9^ Furthermore, SLT may offer comparable outcomes to BLT in select populations. Frail, older adult transplant candidates may be unable to tolerate the increased operative time and physiologic stress of BLT, and as a result, are offered only SLT at some centers.^10^^,^^11^ However, these patients might be at risk for a prolonged course after transplantation if complications develop in the lung allograft such as primary graft dysfunction, infection, or rejection. Additionally, patients with shorter expected lifespans, such as those older than 65 years, may experience a smaller relative benefit from receiving BLT vs SLT because BLT’s advantages become more pronounced over the long term. Despite the potential for comparable outcomes after SLT and BLT in older adult patients, to our knowledge, no recent propensity-matched studies comparing the two procedures have been published. Our study’s aim was to characterize the outcomes of SLT and BLT in older adult recipients in a large contemporary national sample.

## Study Design and Methods

### Population

**Figure 1:**
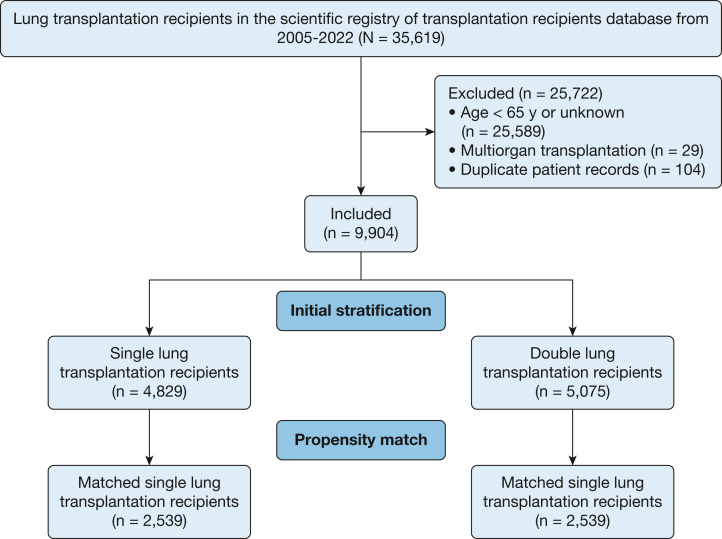
Flow chart displaying the cohorts of patients analyzed in this study.

The Scientific Registry of Transplant Recipients thoracic database was queried for all recipients aged 65 years older who underwent lung transplantation from January 1, 2005, through June 30, 2022. Multiorgan transplant recipients and those with duplicate patient records were excluded (Fig 1). Transplant recipients were stratified into two groups: those undergoing SLT and those undergoing BLT.

A 1:1 nearest-neighbor propensity match was performed to generate matched cohorts from each group. The propensity score model included the following pretransplantation variables: recipient age, sex, BMI, lung disease, lung allocation score, bridging on mechanical ventilation, bridging on extracorporeal membrane oxygenation (ECMO) use, creatinine level, cigarette use history, chronic steroid use, mean pulmonary artery pressure, and Karnofsky performance score, as well as donor age, race, diabetes status, cigarette use history, donor-recipient cytomegalovirus status, and ischemic time. The model used a caliper width of 0.2 times the SD of the propensity score’s logit. Patients were paired 1:1 without replacement. Covariate balance was assessed using standardized mean differences (SMDs), kernel densities, and propensity score histograms. SMDs with an absolute value of ≥ 0.1 were deemed statistically significant.

### Statistical Analysis

Baseline recipient and donor characteristics, as well as recipient morbidity and mortality, are reported for all recipients and were compared between SLT and BLT recipients. Categorical variables are expressed as count (frequency). Continuous variables are presented as median (interquartile range). Comparisons between SLT and BLT groups were performed using χ^2^ tests for categorical variables and Kruskal-Wallis tests for variables with nonparametric distribution. Parametricity was assessed for each continuous variable using the Shapiro-Wilk test. Survival was assessed at 30 days and 1, 3, and 5 years using Kaplan-Meier estimation. Survival comparisons between groups were performed using a log-rank test for unmatched cohorts and a stratified log-rank test for matched cohorts.

A multivariable Cox proportional hazards regression model was performed to determine predictors of 5-year mortality among all recipients included in the propensity match. Univariable prescreening was performed on all variables that were used as covariates in the propensity score model, as well as the variable of SLT vs BLT. Backward stepwise selection was performed on all variables with *P* < .2 on univariable analysis. Adjusted hazard ratios (95% CIs) are presented.

All significance tests were two-tailed. Missing information was managed via exclusion. All statistical analyses were performed using STATA/MP version 17.0 software (StataCorp LLC).

### Ethics

This study was deemed not human participants research on review by the University of Pennsylvania Institutional Review Board (Identifier: 850952; approval date, March 10, 2022), and therefore, no informed consent was required. The study was completed in compliance with the International Society for Heart and Lung Transplantation’s ethics statement.

## Results

### Recipient and Donor Characteristics

From 2005 through 2022, 9,904 adult lung transplantation recipients met inclusion criteria (Fig 1): 4,829 patients (48.8%) underwent SLT and 5,075 patients (51.2%) underwent BLT (Table 1). Propensity matching resulted in 2,539 patients in each cohort with balanced propensity scores and kernel densities (e-Figs 1, 2). On unmatched analysis, 2,533 SLT recipients (52.9%) received a left lung, and on matched analysis, 1,297 SLT recipients (51.1%) received a left lung. No variable in Table 1 showed missingness > 4.0% (e-Fig 3).

**Table 1:** Preoperative Demographics of Older Adult Lung Transplantation Recipients From 2005 Through 2022 Stratified by SLT vs BLT

Variable	Unmatched			Propensity Matched		
	BLT (n = 5,075)	SLT (n = 4,829)	SMD	BLT (n = 2,539)	SLT (n = 2,539)	SMD
Recipient						
Age, y	67 (66-69)	68 (66-71)	**–0.35**	68 (66-70)	67 (66-69)	**0.22**
Female	1,699 (33.5)	1,441 (29.8)	–0.08	785 (30.9)	850 (33.5)	0.05
Ethnicity			0.03			0.06
White	4,354 (85.8)	4,245 (87.9)	—	2,169 (85.4)	2,229 (87.8)	—
Black	275 (5.4)	161 (3.3)	—	118 (4.7)	99 (3.9)	—
Hispanic	301 (5.9)	276 (5.7)	—	175 (6.9)	137 (5.4)	—
BMI, kg/m^2^	26.1 (23.1-28.9)	26.5 (23.6-29.1)	–0.08	26.3 (23.3-29.1)	26.2 (23.3-28.8)	0.05
Diagnosis			**0.23**			–0.05
ILD	3,276 (64.6)	3,591 (74.4)	—	1,748 (68.9)	1,745 (68.7)	—
COPD	1,431 (28.2)	1,085 (22.5)	—	690 (27.2)	662 (26.1)	—
Status at transplantation						
Lung allocation score	40.6 (34.7-53.3)	39.6 (43.7-47.9)	**0.19**	39.5 (34.5-49.3)	40.4 (34.8-50.5)	–0.05
Mechanically ventilated	233 (4.6)	89 (1.8)	**0.16**	54 (2.1)	60 (2.4)	–0.02
Receiving ECMO	122 (2.4)	34 (0.7)	**0.14**	15 (0.6)	25 (1.0)	–0.04
Diabetes	839 (16.6)	871 (18.1)	–0.04	406 (16.1)	461 (18.2)	–0.06
CMV positive	2,828 (57.8)	2,767 (58.7)	–0.02	1,480 (58.3)	1,454 (57.3)	0.02
Cigarette use history	3,605 (71.1)	3,329 (69.0)	0.04	1,793 (70.6)	1,821 (71.7)	–0.03
Chronic steroid use	3,047 (41.2)	2,037 (43.0)	–0.04	1,048 (41.3)	1,063 (41.9)	–0.01
Serum creatinine, mg/dL	0.8 (0.7-1.0)	0.9 (0.7-1.0)	–0.02	0.8 (0.7-1.0)	0.8 (0.7-1.0)	0.05
Serum total bilirubin, mg/dL	0.5 (0.3-0.7)	0.5 (0.3-0.7)	–0.03	0.5 (0.3-0.7)	0.5 (0.3-0.7)	–0.06
Mean pulmonary artery pressure, mm Hg	24 (19-30)	21.7 (17.7-26.7)	**0.36**	22 (17.7-27.3)	23.7 (19.3-28.7)	–**0.14**
Karnofsky performance score	50 (40-60)	50 (40-60)	–**0.10**	50 (40-60)	50 (40-60)	–0.02
Donor or transplant						
Age, y	34 (24-48)	34 (23-47)	0.04	34 (24-47)	34 (23-47)	0.00
Female	1,991 (39.2)	1,717 (35.6)	–0.08	996 (39.2)	917 (36.1)	–0.06
Ethnicity			–0.05			0.03
White	3,198 (63.0)	2,850 (59.0)		1,537 (60.5)	1,531 (60.3)	—
Black	844 (16.6)	925 (18.6)		420 (16.5)	472 (18.6)	—
Hispanic	821 (16.2)	896 (18.6)		465 (18.3)	452 (17.8)	—
BMI, kg/m^2^	25.8 (22.8-29.7)	25.4 (22.6-29.1)	0.07	25.8 (22.8-29.6)	25.3 (22.5-29.1)	0.07
Diabetes	431 (8.5)	366 (7.6)	0.03	206 (8.1)	201 (7.9)	0.01
CMV positive	3,121 (61.7)	3,017 (62.6)	–0.02	1,566 (61.7)	1,565 (61.6)	0.00
CMV mismatch^a^	1,256 (25.8)	1,171 (24.9)	0.02	637 (25.1)	646 (25.4)	–0.00
Cigarette use history	460 (9.2)	398 (8.4)	0.03	210 (8.3)	218 (8.6)	–0.01
Serum creatinine, mg/dL	1.0 (0.7-1.5)	1.0 (0.8-1.5)	0.01	1.0 (0.7-1.5)	1.0 (0.8-1.5)	0.02
Purulent secretions on bronchoscopy	875 (17.9)	814 (17.6)	0.01	475 (19.2)	433 (17.7)	0.04
Ischemic time, h	5.8 (4.6-6.7)	4.2 (3.5-5.1)	**0.73**	5.0 (4.2-6.0)	4.8 (4.0-5.6)	0.07
Left lung only	—	2,553 (52.9)		—	1,297 (51.1)	—

Data are presented as No. (%) or are medians (interquartile range) unless otherwise indicated. Boldface values indicate statistical significance. Em dashes indicate no SMD was assessed to compare subgroups within variable. BLT = bilateral lung transplantation; CMV = cytomegalovirus; ECMO = extracorporeal membrane oxygenation; ILD = interstitial lung disease; SMD = standardized mean difference; SLT = single lung transplantation.

a Donor showed positive results for CMV and recipient showed negative results.

On unmatched analysis, the median ages of patients undergoing SLT and BLT were 68 and 67 years, respectively (SMD = –0.35). Patients who underwent SLT showed slightly lower median lung allocation scores (39.6 vs 40.6; SMD = 0.19), higher rates of interstitial lung disease (74.4% vs 64.6%; SMD = 0.23), and lower rates of preoperative ECMO (0.7% vs 2.4%; SMD = 0.14) and mechanical ventilation (1.8% vs 4.6%; SMD = 0.16) than patients undergoing BLT. Groups did not differ with respect to recipient sex, BMI, serum creatinine level, or any donor characteristics (SMD < |0.1| for all). However, as expected, patients who underwent SLT experienced lower graft ischemic times (4.2 hours vs 5.8 hours; SMD = 0.73).

After propensity matching, patients who underwent SLT had a slightly lower median age than patients who underwent BLT (67 years vs 68 years; SMD = 0.23) and slightly higher mean pulmonary artery pressure (23.7 mm Hg vs 22 mm Hg; SMD = –0.14). However, no other measured recipient or donor characteristics differed significantly between groups after propensity matching (SMD < |0.1| for all) (Table 1).

### Morbidity

At 72 hours after transplantation, on unmatched analysis, patients who underwent SLT were less likely than those who underwent BLT to be intubated (16.6% vs 31.4%; *P* < .001) or require inhaled nitric oxide (4.6% vs 9.3%; *P* < .001) and were equally likely to receive ECMO (4.9% vs 5.9%; *P* = .091) (Table 2). Patients who underwent SLT also were less likely to be reintubated (14.7% vs 22.3%; *P* < .001) or to require dialysis (3.9% vs 8.2%; *P* < .001) during the index hospital stay and experienced significantly shorter median hospital lengths of stay (14 days vs 20 days; *P* < .001). However, patients who underwent SLT showed higher rates of both acute rejection (7.3% vs 6.0%; *P* = .010) and rejection requiring treatment within 1 year of transplantation (25.4% vs 18.5%; *P* < .001) than patients undergoing BLT.

**Table 2:** Morbidity of Older Adult Lung Transplantation Recipients From 2005 Through 2022 Stratified by SLT vs BLT

Variable	Unmatched			Propensity Matched		
	BLT	SLT	*P* Value	BLT	SLT	*P* Value
72 h after transplantation						
Intubated	1,109 (31.4)	458 (16.6)	**< .001**	513 (27.3)	284 (20.2)	**< .001**
Receiving ECMO	208 (5.9)	135 (4.9)	.091	79 (4.2)	85 (6.1)	**.014**
Pao_2_ to Fio_2_ ratio	300 (215-397)	280 (200-362)	**< .001**	300 (212-396)	281 (205-375)	**.004**
< 300	978 (19.3)	681 (14.1)	—	508 (20.0)	349 (13.8)	—
200-300	587 (11.6)	398 (8.2)	—	292 (11.5)	208 (8.2)	—
< 200	407 (8.0)	296 (6.1)	—	225 (8.9)	147 (5.8)	—
Receiving inhaled NO	327 (9.3)	124 (4.6)	**< .001**	144 (7.7)	88 (6.4)	.148
Before discharge						
Intubation ≥ 5 d	956 (24.8)	589 (14.2)	**< .001**	480 (23.7)	336 (15.5)	**< .001**
Reintubation	1,111 (22.3)	701 (14.7)	**< .001**	499 (19.8)	371 (14.7)	**< .001**
Acute rejection	302 (6.0)	351 (7.3)	**.010**	144 (5.7)	191 (7.5)	**.008**
Dialysis	407 (8.2)	187 (3.9)	**< .001**	163 (6.4)	106 (4.2)	**< .001**
Stroke	171 (3.4)	79 (1.7)	**< .001**	74 (2.9)	40 (1.6)	**.001**
Overall						
Hospital length of stay	20 (13-33)	14 (10-22)	**< .001**	18 (13-29)	14 (10-23)	**< .001**
Airway dehiscence	103 (2.0)	49 (1.0)	**< .001**	48 (1.9)	29 (1.2)	**.029**
Rejection (treated within 1 y of transplantation)	729 (18.5)	961 (25.4)	**< .001**	342 (17.7)	561 (27.5)	**< .001**

Data are presented as No. (%) or are medians (interquartile range) unless otherwise indicated. Boldface values indicate statistical significance. Em dashes indicate no *P* value was calculated for subgroup within variable. BLT = bilateral lung transplantation; ECMO = extracorporeal membrane oxygenation; NO = nitric oxide; SLT = single lung transplantation.

On matched analysis, patients who underwent SLT again were less likely to be intubated 72 hours after transplantation (20.2% vs 27.3%; *P* < .001), showed lower rates of reintubation (14.7% vs 19.8%; *P* < .001) and dialysis use (4.2% vs 6.4%; *P* < .001) during the index hospital stay, and experienced shorter lengths of stay (14 days vs 18 days; *P* < .001). Additionally, after matching, patients who underwent SLT showed higher rates of acute rejection (7.5% vs 5.7%; *P* = .008) and rejection requiring treatment within 1 year of transplantation (27.5% vs 17.7%; *P* < .001).

Missingness was considerable (> 10%) for morbidity variables including rates of intubation, inhaled nitric oxide use, and ECMO use at 72 hours after transplantation, as well as rates of rejection requiring treatment within 1 year of transplantation (e-Fig 4). Missingness was low (< 3.5%) for variables including rates of reintubation, acute rejection, and dialysis need, as well as hospital length of stay.

### Mortality

**Figure 2:**
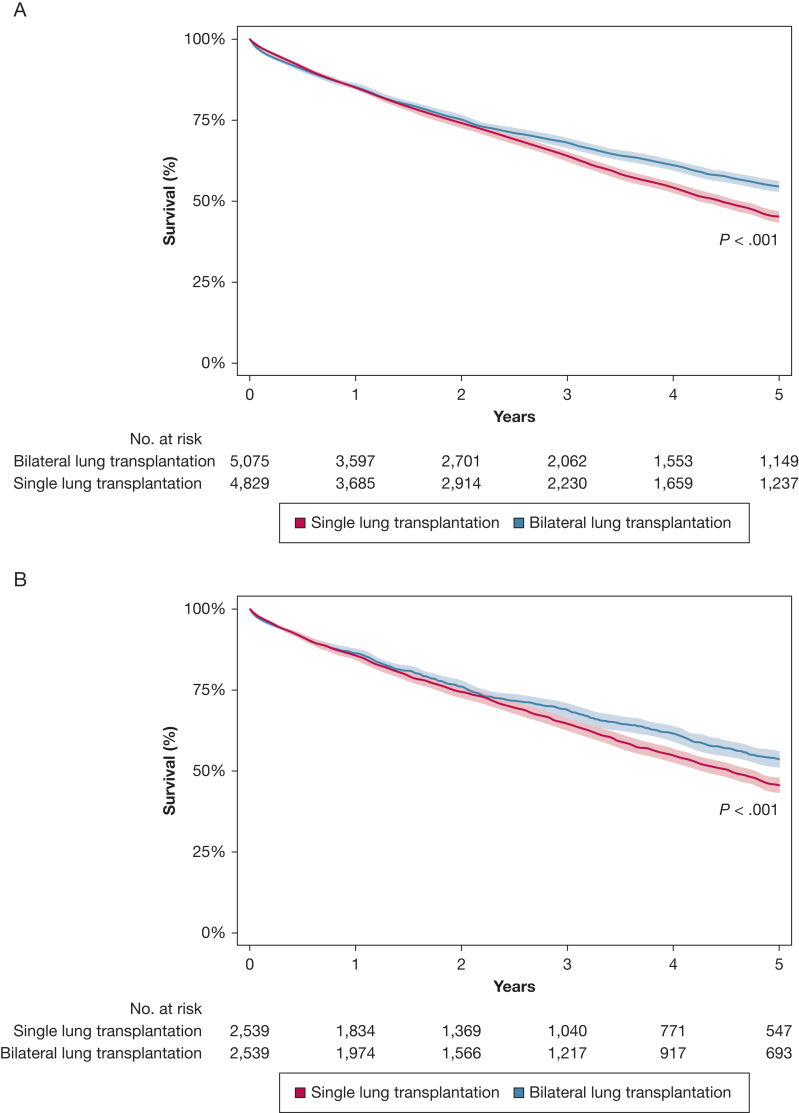
A, B, Kaplan-Meier curves showing 5-year survival among older adult lung transplant recipients from 2005 through 2022, stratified by single versus bilateral lung transplant on unmatched analyses (A) and propensity-matched anaylses (B).

For the overall cohort of recipients, survival at 30 days and 1, 3, and 5 years was 97.4%, 85.2%, 65.8%, and 49.2%, respectively. On unmatched analysis, patients who underwent SLT showed slightly higher 30-day survival (97.8% vs 97.0%; *P* = .009), comparable 1-year survival (85.0% vs 85.3%; *P* = .818), and lower 3-year survival (63.9% vs 68.0%; *P* = .003) and 5-year survival (45.0% vs 54.3%; *P* < .001) (Table 3, Fig 2). On matched analysis, no significant differences were found in 30-day or 1-year survival, but patients who underwent SLT showed lower 3-year survival (64.5% vs 68.7%; *P* = .019) and 5-year survival (44.6% vs 53.1%; *P* < .001). Lower 5-year survival among patients who underwent SLT also was found when limiting analysis to recipients with COPD (46.8% vs 55.0%; *P* = .003), recipients with interstitial lung disease (44.5% vs 54.1%; *P* < .001), and recipients from 2012 through 2022 (47.2% vs 55.1%; *P* < .001). Relative to patients who underwent SLT of the left lung, patients who underwent SLT of the right lung showed higher 5-year survival (47.3% vs 43.0%; *P* = .004), but comparable 1-year survival (85.4% vs 84.6%; *P* = .415) and 30-day survival (98.0% vs 97.6%; *P* = .415). Missingness of survival data was < 2.0% at every time point on unmatched analysis and 0% on matched analysis (e-Fig 5).

**Table 3:** Survival and Causes of Death Among Older Adult Lung Transplantation Recipients From 2005 Through 2022 Stratified by SLT vs BLT

Variable	Unmatched			Propensity Matched		
	BLT	SLT	*P* Value	BLT	SLT	*P* Value
Survival						
30 d	97.0% (96.5%-97.4%)	97.8% (97.3%-98.2%)	**.009**	97.3% (96.5%-97.8%)	97.6% (96.9%-98.1%)	.414
1 y	85.3% (84.3%-86.3%)	85.0% (83.9%%-86.0%)	.818	86.3% (84.9%-87.6%)	85.5% (84.0%-86.8%)	.496
3 y	68.0% (66.5%-69.5%)	63.9% (62.4%-65.3%)	**.003**	68.7% (66.6%-70.7%)	64.5% (62.5%-66.5%)	**.019**
5 y	54.3% (52.5%-56.0%)	45.0% (43.3%-46.6%)	**< .001**	53.4% (50.9%-55.9%)	45.4% (43.1%-47.6%)	**< .001**
Cause of death			**< .001**			**< .001**
Infection	447 (24.4%)	549 (23.3%)	—	219 (24.9%)	294 (22.8%)	—
Malignancy	233 (12.7%)	408 (17.3%)	—	121 (13.7%)	219 (17.0%)	—
Rejection	229 (12.5%)	319 (13.5%)	—	114 (12.9%)	186 (14.4%)	—
Acute graft failure	67 (3.7%)	58 (2.5%)	—	26 (3.0%)	29 (2.3%)	—
Other pulmonary	330 (18.0%)	531 (22.5%)	—	154 (17.5%)	298 (23.1%)	—
Cardiovascular	191 (10.4%)	212 (9.0%)	—	101 (11.5%)	115 (8.9%)	—
Cerebrovascular	97 (5.3%)	69 (2.9%)	—	47 (5.3%)	37 (2.9%)	—
Hemorrhage	34 (1.9%)	44 (1.9%)	—	13 (1.5%)	22 (1.7%)	—
Multisystem organ failure	117 (6.4%)	98 (4.2%)	—	47 (5.3%)	56 (4.3%)	—
Other	86 (4.7%)	73 (3.1%)	—	39 (4.4%)	35 (2.7%)	—

Data are presented as No. (%) or Kaplan-Meier survival function (95% CI) unless otherwise indicated. Boldface values indicate statistical significance. Em dashes indicate no *P* value was calculated for subgroup within variable. BLT = bilateral lung transplantation; SLT = single lung transplantation.

The most frequent cause of death in both patients who underwent BLT and those who underwent SLT on both unmatched and matched analyses was infection (Table 3). Rejection accounted for a similar proportion of deaths in BLT and SLT recipients (12.5% vs 13.5%, respectively, on unmatched analysis; 12.9% vs 14.4%, respectively, on matched analysis). Missingness of cause of death data was approximately 15% on both unmatched and matched analyses (e-Fig 5).

A Cox regression assessing predictors of 5-year mortality among propensity-matched older adult lung transplant recipients found SLT to be a significant predictor of mortality, with an adjusted hazard ratio (aHR) of 1.19 (*P* < .001) on both univariable and multivariable analysis (Table 4). Other predictors of 5-year mortality on multivariable analysis included: recipient BMI ≥ 30 kg/m^2^ (aHR, 1.13; *P* = .007), ECMO use at time of transplantation (aHR, 2.12; *P* < .001), mean pulmonary artery pressure ≥ 30 mm Hg (aHR, 1.26; *P* < .001), Karnofsky performance score of < 60 (aHR, 1.12; *P* = .014), donor of Black race (aHR, 1.19; *P* = .002), donor with diabetes (aHR, 1.24; *P* = .007), and donor and recipient cytomegalovirus mismatch (aHR, 1.21; *P* < .001). Additional multivariable Cox regressions for 5-year mortality that replaced the covariate SLT vs BLT with left lung SLT vs BLT and right lung SLT vs BLT found that left lung SLT (aHR, 1.27; *P* < .001) and right lung SLT (aHR, 1.13; *P* = .031) each were independent risk factors for 5-year mortality (e-Figs 6, 7).

**Table 4:** Cox Regression for 5-Year Mortality Among Propensity-Matched Older Adult Lung Transplantation Recipients From 2005 Through 2022

Variable	Univariable Regression		Multivariable Regression	
	Adjusted Hazard Ratio(95% CI)	*P* Value	Adjusted Hazard Ratio (95% CI)	*P* Value
Operation				
SLT (vs DLT)	1.19 (1.09-1.30)	**< .001**	1.19 (1.09-1.30)	**< .001**
Recipient characteristics				
Black race	0.91 (0.72-1.13)	.395	—	—
BMI ≥ 30 kg/m^2^	1.13 (1.00-1.27)	**.046**	1.13 (1.00-1.27)	**.007**
Lung allocation score ≥ 75	1.16 (0.37-3.59)	.802	—	—
Mechanically ventilated at time of transplantation	1.06 (0.80-1.41)	.692	—	—
Receiving ECMO at time of transplantation	2.02 (1.31-3.13)	**.002**	2.12 (1.42-3.15)	**< .001**
Cigarette use history	1.02 (0.92-1.12)	.731	—	—
Chronic steroid history	1.14 (1.04-1.25)	**.004**	1.14 (1.04-1.25)	**.004**
Serum creatinine ≥ 2 mg/dL	1.40 (0.77-2.54)	.267	—	—
Mean pulmonary artery pressure ≥ 30 mm Hg	1.26 (1.14-1.41)	**< .001**	1.26 (1.13-1.40)	**< .001**
Karnofsky performance score < 60	1.12 (1.02-1.22)	**.016**	1.12 (1.02-1.23)	**.014**
Donor or transplant characteristics				
Age ≥ 50 y	1.08 (0.97-1.20)	.183	1.08 (0.97-1.20)	.169
Black race	1.19 (1.07-1.33)	**.002**	1.19 (1.06-1.33)	**.002**
Diabetes	1.24 (1.05-1.45)	**.009**	1.24 (1.06-1.46)	**.007**
Cigarette use history	1.09 (0.93-1.27)	.287	—	—
CMV mismatch^a^	1.21 (1.09-1.33)	**< .001**	1.21 (1.09-1.33)	**< .001**
Ischemic time ≥ 6 h	1.06 (0.95-1.18)	.319	—	—

Boldface values indicate statistical significance. Em dashes indicate variable did not meet inclusion criteria to be in a multivariable regression. BLT = bilateral lung transplantation; CMV = cytomegalovirus; ECMO = extracorporeal membrane oxygenation; SLT = single lung transplantation.

a Donor showed positive results for CMV and recipient showed negative results.

## Discussion

Our study examined the morbidity and mortality of older adult lung transplantation recipients in the United States over a 17-year period and found that SLT recipients demonstrated decreased postoperative morbidity and comparable early mortality relative to BLT recipients. Regarding postoperative morbidity, SLT recipients showed lower rates of reintubation and dialysis use after transplantation, as well as significantly shorter hospital lengths of stay. Regarding early mortality, SLT and BLT recipients showed comparable 1-year survival. However, SLT recipients showed significantly higher rates of graft rejection and lower 5-year survival than BLT recipients.

It is unsurprising that SLT recipients fared better in the early postoperative period than BLT recipients. SLT is a shorter and technically simpler operation with decreased graft ischemic time.^12^^,^^13^ Previous studies also have found that SLT requires less frequent intraoperative ECMO support.^14^ Additionally, because pulmonary infection and severe pulmonary hypertension before transplantation often are considered contraindications to SLT, it is possible that SLT recipients are at baseline healthier than BLT recipients.^12^ Our study confirmed that in older adult patients, SLT results in less early postoperative morbidity, even after matching patients with respect to recipient characteristics, donor characteristics, and graft ischemic time. Consistent with previous studies that focused on younger cohorts of lung transplant recipients, ours also found little difference in mortality at 30 days and 1 year between SLT and BLT recipients.^2^^,^^3^ These findings suggest that SLT is a reasonable operation to offer to older adult patients.

It is worth noting that our study also identified two disadvantages of SLT relative to BLT: increased rejection rates and worsened long-term survival. Although BLT’s long-term survival advantage already has been demonstrated in younger cohorts,^1^, ^2^, ^3^^,^^15^ the association between transplant type on rejection rates is less robust. Studies comparing adult SLT and BLT recipients of all ages generally have found an association between SLT and bronchiolitis obliterans syndrome, but few have linked SLT to acute rejection.^12^^,^^15^^,^^16^ It is unclear why SLT is associated with higher rates of both acute rejection and episodes of rejection requiring treatment within 1 year of transplantation. The clinical significance of these findings is opaque because the rates of death resulting from rejection were similar in the unmatched and matched cohorts. Additionally, the Scientific Registry of Transplant Recipients database does not provide granular data regarding acute rejection type (humoral vs acute cellular) and pathologic grading, or chronic lung allograft dysfunction subtypes and rates. That said, this finding must be taken into consideration when determining which patients are appropriate candidates for SLT versus BLT. The long-term survival difference between BLT and SLT recipients also must factor into this decision heavily. Although a statistically significant difference in survival between these two operations at 3 years was found, the absolute survival difference at 3 years is about 4% and then grows to 8% to 9% at 5 years. Notably, as previously reported, right lung SLT recipients show greater 5-year survival than left lung recipients, but both right and left lung SLT are independent risk factors for 5-year mortality on regression analysis.^17^ Whether these worse long-term outcomes for SLT recipients justify the expected population-level outcomes of performing SLT—that is, more patients undergoing transplantation and decreased waitlist-associated morbidity and mortality—remains a judgment call that individual providers, transplant teams, and guideline-writing committees must make.

Although our study offers insight into the morbidity and mortality that older adult lung transplantation recipients can expect after SLT or BLT, its conclusions are inherently limited by the study’s retrospective design and use of a single, national database. Patients in this study were not randomized to receive BLT or SLT, implying that their transplant teams offered whichever transplant they deemed most appropriate. Additionally, the Scientific Registry of Transplant Recipients database used in this study contains data on all lung transplants conducted in the United States from 2005 through 2022, so its findings may not generalize as well to patients outside of the United States. Furthermore, this study lacks data on critically important postoperative outcomes such as primary graft dysfunction rates, spirometry values, functional outcomes, and health-related quality-of-life scores. It is possible that although the survival benefits of BLT over SLT are modest, these other benefits are more profound. Some of the postoperative outcomes examined in the study—for example, incidence of rejection requiring treatment within 1 year of transplantation and cause of death—showed a high degree of missingness, and therefore any association between SLT and these outcomes should be interpreted with significant caution. Finally, this study examined only the individual outcomes of patients who underwent BLT or SLT, and did not assess the population-level effects of offering SLT vs BLT in older adult patients.

## Interpretation

Our study demonstrated acceptable outcomes for SLT in older adult patients: lower postoperative respiratory and renal complications than BLT, with comparable early mortality, increased rejection rates, and modest decrements in 3-year and 5-year survival. Considering these data, decisions to perform SLT for older adult patients are reasonable, but still must be made on a case-by-case basis by a multidisciplinary team. National and institutional guidelines should consider the outcomes reported within this study, as well as SLT’s potential benefits for improving outcomes among a wider subset of waitlisted patients.

## Funding/Support

The authors have reported to *CHEST* that no funding was received for this study.

## Financial/Nonfinancial Disclosures

None declared.

## Supplementary Data

e-Online Data
