# Supplementary material for: Single vs Double Lung Transplantation in Older Adults: A Propensity-Matched Analysis
Source: Chest. 2024 Sep 5;167(2):518–28. doi: 10.1016/j.chest.2024.08.044 (PMC11867891; doi:10.1016/j.chest.2024.08.044)
Supplement: e-Online Data [file mmc1.docx]

**Supplementary Figures with Legends**

e-Figure 1. Histogram of propensity scores of older adult lung transplant recipients (A) before and (B) after excluding patients who were not successfully matched.

**(A)**

**(B)**

e-Figure 2. Kernel densities of older adult lung transplant recipients before (A) and after (B) excluding patients who were not successfully matched

**(A)**

**(B)**

e-Figure 3. Missingness of variables related to pre-transplant demographics of older adult lung transplant recipients from 2005-2022, stratified by single versus bilateral lung transplant.

|  | Unmatched | | Propensity-Matched | |
| --- | --- | --- | --- | --- |
|  | BLT  (n=5,075) | SLT  (n=4,829) | BLT  (n=2,539) | SLT  (n=2,539) |
| **Recipient** |  |  |  |  |
| Age, y | 0 (0%) | 0 (0%) | 0 (0%) | 0 (0%) |
| Female | 0 (0%) | 0 (0%) | 0 (0%) | 0 (0%) |
| Ethnicity | 0 (0%) | 0 (0%) | 0 (0%) | 0 (0%) |
| BMI, m/kg^2^ | 0 (0%) | 0 (0%) | 0 (0%) | 0 (0%) |
| Diagnosis | 6 (0.1%) | 3 (0.1%) | 0 (0%) | 0 (0%) |
| Status at transplant |  |  |  |  |
| *Lung allocation score* | 10 (0.2%) | 31 (0.6%) | 0 (0%) | 0 (0%) |
| *Mechanically ventilated* | 0 (0%) | 0 (0%) | 0 (0%) | 0 (0%) |
| *On ECMO* | 0 (0%) | 0 (0%) | 0 (0%) | 0 (0%) |
| Diabetes | 23 (0.5%) | 19 (0.4%) | 9 (0.4%) | 4 (0.2%) |
| CMV positive | 183 (3.6%) | 118 (2.4%) | 0 (0%) | 0 (0%) |
| Cigarette use history | 7 (0.1%) | 7 (0.1%) | 0 (0%) | 0 (0%) |
| Chronic steroid use | 106 (2.1%) | 95 (2.0%) | 0 (0%) | 0 (0%) |
| Serum creatinine, mg/dL | 67 (1.3%) | 53 (1.1%) | 0 (0%) | 0 (0%) |
| Serum total bilirubin, mg/dL | 74 (1.5%) | 86 (1.8%) | 3 (0.1%) | 12 (0.5%) |
| Mean pulmonary artery pressure, mmHg | 198 (3.9%) | 184 (3.8%) | 0 (0%) | 0 (0%) |
| Karnofsky performance score | 140 (2.8%) | 114 (2.4%) | 0 (0%) | 0 (0%) |
|  |  |  |  |  |
| **Donor / transplant** |  |  |  |  |
| Age, y | 0 (0%) | 0 (0%) | 0 (0%) | 0 (0%) |
| Female | 0 (0%) | 0 (0%) | 0 (0%) | 0 (0%) |
| Ethnicity | 0 (0%) | 0 (0%) | 0 (0%) | 0 (0%) |
| BMI, m/kg^2^ | 5 (0.1%) | 2 (0.0%) | 3 (0.1%) | 2 (0.1%) |
| Diabetes | 28 (0.6%) | 30 (0.6%) | 0 (0%) | 0 (0%) |
| CMV positive | 20 (0.4%) | 12 (0.3%) | 0 (0%) | 0 (0%) |
| CMV mismatch | 203 (4.0%) | 129 (2.7%) | 0 (0%) | 0 (0%) |
| Cigarette use history | 80 (1.6%) | 79 (1.6%) | 0 (0%) | 0 (0%) |
| Serum creatinine, mg/dL | 28 (0.6%) | 11 (0.2%) | 8 (0.3%) | 0 (0%) |
| Purulent secretions on bronchoscopy | 185 (3.7%) | 193 (4.0%) | 65 (2.6%) | 98 (3.9%) |
| Ischemic time, h | 104 (2.1%) | 129 (2.7%) | 0 (0%) | 0 (0%) |
| Left lung only | - | 0 (0%) | - | 0 (0%) |

*All values are counts (percentage). BLT = bilateral lung transplant. BMI = body mass index. CMV = cytomegalovirus. CMV mismatch = donor positive for CMV and recipient negative. COPD = chronic obstructive pulmonary disease. ECMO = extracorporeal membrane oxygenation. ILD = interstitial lung disease. SMD = standardized mean difference. SLT = single lung transplant.*

e-Figure 4. Missingness of variables related to morbidity of older adult lung transplant recipients from 2005-2022, stratified by single versus bilateral lung transplant.

|  | Unmatched | | | Propensity-Matched | |
| --- | --- | --- | --- | --- | --- |
|  | BLT  (n=5,075) | | SLT  (n=4,829) | BLT  (n=2,539) | SLT  (n=2,539) |
| **72 hours post-transplant** |  |  | |  |  |
| Intubated | 1,547 (30.5%) | 2,076 (43.0%) | | 658 (25.9%) | 1,135 (44.7%) |
| On ECMO | 1,546 (30.5%) | 2,083 (43.1%) | | 655 (25.8%) | 1,142 (45.0%) |
| P/F ratio | 1,984 (39.1%) | 1,200 (24.9%) | | 1,511 (59.5%) | 1,909 (75.2%) |
| On inhaled NO | 1,561 (39.8%) | 2,106 (43.6%) | | 663 (26.1%) | 1,155 (45.5%) |
|  |  |  | |  |  |
| **Prior to discharge** |  |  | |  |  |
| Intubation ≥5 d | 1,215 (23.9%) | 666 (13.8%) | | 510 (20.1%) | 365 (14.4%) |
| Reintubation | 95 (1.9%) | 74 (1.5%) | | 13 (0.5%) | 12 (0.5%) |
| Acute rejection | 79 (1.6%) | 49 (1.0%) | | 6 (0.2%) | 3 (0.1%) |
| Dialysis | 90 (1.8%) | 57 (1.2%) | | 8 (0.3%) | 5 (0.2%) |
| Stroke | 111 (2.2%) | 73 (1.5%) | | 14 (0.6%) | 17 (0.7%) |
|  |  |  | |  |  |
| **Overall** |  |  | |  |  |
| Hospital length of stay | 170 (3.4%) | 107 (2.2%) | | 43 (1.7%) | 25 (1.0%) |
| Airway dehiscence | 101 (2.0%) | 78 (1.6%) | | 14 (0.6%) | 15 (0.6%) |
| Rejection (treated w/in year of transplant) | 1,333 (26.3%) | 1,041 (21.6%) | | 607 (23.9%) | 495 (19.5%) |

*All values are counts (percentage). BLT = bilateral lung transplant. ECMO = extracorporeal membrane oxygenation. NO = nitric oxide. P/F ratio = ratio of arterial oxygen partial pressure to fraction of inspired oxygen. SLT = single lung transplant.*

e-Figure 5. Missingness of variables related to survival and causes of death among older adult lung transplant recipients from 2005-2022, stratified by single versus bilateral lung transplant.

| Variable | Unmatched | | Propensity-matched | |
| --- | --- | --- | --- | --- |
|  | BLT | SLT | BLT | SLT |
| **Survival** |  |  |  |  |
| 30-day | 6 (0.1%) | 14 (0.3%) | 0 (0%) | 0 (0%) |
| One-year | 15 (0.3%) | 40 (0.8%) | 0 (0%) | 0 (0%) |
| Three-year | 27 (0.5%) | 82 (1.7%) | 0 (0%) | 0 (0%) |
| Five-year | 33 (0.7%) | 96 (2.0%) | 0 (0%) | 0 (0%) |
|  |  |  |  |  |
| **Cause of death** | 337 (15.6%) | 426 (15.3%) | 180 (17.0%) | 221 (14.6%) |

*All values are counts (percentage). BLT = bilateral lung transplant. SLT = single lung transplant.*

e-Figure 6. Cox regression for five-year mortality among propensity matched older adult lung transplant recipients from 2005-2022 that includes left lung only single lung transplant as an additional covariate

| Variable | Univariable regression | | Multivariable regression | |
| --- | --- | --- | --- | --- |
|  | Adjusted hazard ratio  [95% CI] | P value | Adjusted hazard ratio [95% CI] | P value |
| **Operation** |  |  |  |  |
| Left lung SLT (vs. double lung transplant) | 1.27  [1.14-1.41] | **<0.001** | 1.27  [1.14-1.41] | **<0.001** |
| **Recipient characteristics** |  |  |  |  |
| Black race | 0.92  [0.72-1.19] | 0.533 | - | - |
| Body mass index ≥30 kg/m^2^ | 1.16  [1.02-1.33] | **0.028** | 1.17  [1.02-1.34] | **0.024** |
| Lung allocation score ≥75 | 1.51  [0.38-6.06] | 0.560 | - | - |
| Mechanically ventilated at time of transplant | 0.89  [0.62-1.28] | 0.541 | - | - |
| On ECMO at time of transplant | 2.18  [1.31-3.62] | **0.003** | 2.05  [1.28-3.28] | **0.003** |
| Cigarette use history | 0.98  [0.44-2.19] | 0.957 | - | - |
| Chronic steroid history | 1.15  [1.04-1.28] | **0.009** | 1.15  [1.03-1.27] | **0.011** |
| Serum creatinine ≥2 mg/dL | 0.98  [0.44-2.19] | 0.957 | - | - |
| Mean pulmonary artery pressure ≥30 mmHg | 1.24  [1.09-1.41] | **0.001** | 1.23  [1.09-1.40] | **0.001** |
| Karnofsky performance score <60 | 1.09  [0.98-1.21] | 0.104 | 1.09  [0.98 -1.21] | 0.110 |
| **Donor / transplant characteristics** |  |  |  |  |
| Age ≥50 years | 1.04  [0.91-1.18] | 0.579 | - | - |
| Black race | 1.23  [1.08-1.41] | **0.002** | 1.23  [1.08-1.40] | **0.002** |
| Diabetes | 1.23  [1.02-1.49] | **0.034** | 1.25  [1.04-1.50] | **0.020** |
| Cigarette use history | 1.17  [0.98-1.40] | 0.077 | 1.17  [0.98-1.40] | 0.080 |
| CMV mismatch | 1.21  [1.08-1.36] | **0.001** | 1.22  [1.08-1.37] | **0.001** |
| Ischemic time ≥6 hours | 1.05  [0.93-1.20] | 0.418 | - | - |

*All values other than p values are adjusted hazard ratios [95% confidence interval]. Bold p values are statistically significant. CI = confidence interval. CMV mismatch = donor positive for cytomegalovirus and recipient negative. ECMO = extracorporeal membrane oxygenation. SLT = single lung transplant.*

e-Figure 7. Cox regression for five-year mortality among propensity matched older adult lung transplant recipients from 2005-2022 that includes right lung only single lung transplant as an additional covariate

| Variable | Univariable regression | | Multivariable regression | |
| --- | --- | --- | --- | --- |
|  | Adjusted hazard ratio  [95% CI] | P value | Adjusted hazard ratio [95% CI] | P value |
| **Operation** |  |  |  |  |
| Right lung SLT (vs. double lung transplant) | 1.13  [1.00-1.26] | **0.033** | 1.13  [1.01-1.26] | **0.031** |
| **Recipient characteristics** |  |  |  |  |
| Black race | 0.87  [0.67-1.14] | 0.323 | - | - |
| Body mass index ≥30 kg/m^2^ | 1.19  [1.03-1.36] | **0.015** | 1.19  [1.04-1.37] | **0.013** |
| Lung allocation score ≥75 | 0.55  [0.18-1.70] | 0.297 | - | - |
| Mechanically ventilated at time of transplant | 1.11  [0.80-1.55] | 0.530 | - | - |
| On ECMO at time of transplant | 1.56  [0.88-2.76] | 0.125 | 1.69  [1.01-2.82] | **0.046** |
| Cigarette use history | 0.99  [0.88-1.12] | 0.925 | - | - |
| Chronic steroid history | 1.12  [1.00-1.25] | **0.033** | 1.12  [1.01-1.25] | **0.036** |
| Serum creatinine ≥2 mg/dL | 1.80  [0.92-3.50] | 0.084 | 1.81  [0.94-3.50] | 0.077 |
| Mean pulmonary artery pressure ≥30 mmHg | 1.28  [1.12-1.45] | **<0.001** | 1.27  [1.12-1.44] | **<0.001** |
| Karnofsky performance score <60 | 1.14  [1.02-1.28] | **0.017** | 1.15  [1.03-1.28] | **0.013** |
| **Donor / transplant characteristics** |  |  |  |  |
| Age ≥50 years | 1.14  [1.00 -1.30] | **0.045** | 1.14  [1.00 -1.30] | **0.042** |
| Black race | 1.20  [1.05-1.37] | **0.008** | 1.19  [1.04-1.36] | **0.011** |
| Diabetes | 1.24  [1.03-1.49] | **0.024** | 1.24  [1.03-1.49] | **0.022** |
| Cigarette use history | 1.07  [0.893-1.28] | 0.475 | - | - |
| CMV mismatch | 1.16  [1.03-1.31] | **0.015** | 1.16  [1.03-1.31] | **0.014** |
| Ischemic time ≥6 hours | 1.04  [0.91-1.19] | 0.561 | - | - |

*All values other than p values are adjusted hazard ratios [95% confidence interval]. Bold p values are statistically significant. CI = confidence interval. CMV mismatch = donor positive for cytomegalovirus and recipient negative. ECMO = extracorporeal membrane oxygenation. SLT = single lung transplant.*
